# Case Report: A papillary thyroid microcarcinoma patient with skip lymph node metastasis and multiple distant metastasis

**DOI:** 10.3389/fsurg.2022.1019846

**Published:** 2023-01-18

**Authors:** Qin Jiang, Mimi Zhai, Xiang Lin, Chutong Ren, Yunxia Li, Fei Ye, Yi Gong, Sushun Liu

**Affiliations:** ^1^ Department of General Surgery, The Second Xiangya Hospital, Central South University, Changsha, China; ^2^ Xiangya Nursing School, Central South University, Changsha, China; ^3^ Department of General Surgery, Huaihua Second People’s Hospital, Huaihua, China

**Keywords:** papillary thyroid microcarcinoma, skip lymph node metastasis, shoulder metastasis, distant metastasis, case report

## Abstract

Papillary thyroid carcinoma (PTC) is the most common type of thyroid cancer. Papillary thyroid microcarcinoma (PTMC) is defined as PTC with a diameter less than 1 centimeter. Most lymph nodes of PTC patients have metastasized to the central neck, and a few lymph nodes have metastasized to the lateral neck. Skip lymph node metastasis, that is, lateral cervical lymph node metastasis without central lymph node metastasis, is even less common. Additionally, distant metastasis of PTMC is also rare, mainly occurring in the lung and bone. Here, we reported a case of PTMC patient with skip lymph node metastasis and multiple distant metastasis. The patient presented with a huge shoulder mass and the primary tumor was found to originate from the thyroid. However, the patient only suffered with PTMC *via* postoperative pathological results, and interestingly, the patient only had skip lymph node metastasis. Thus, we should focus on PTMC patients with lateral cervical lymph nodes metastasis, especially those with skip metastasis. In addition, this case provides a new perspective for us to understand of skip lymph metastasis and distant metastasis of PTMC.

## Established facts

Papillary thyroid carcinoma (PTC) is the most common endocrine malignant tumor with an increasing incidence.

The incidence of distant metastasis of PTC is less than 5%. The main locations of distant metastases are the pulmonary and bone.

## Novel insights

For suspected metastatic lymph nodes, fine needle aspiration cytology (FNAC) combined with the thyroglobulin might be useful.

Inflammation caused by residual sutures is also a factor should not be ignored in thyroid cancer.

## Introduction

Thyroid cancer is the most common endocrine malignant tumor, and its incidence rate is rising. Papillary thyroid carcinoma (PTC) is the most common thyroid carcinoma. Papillary thyroid microcarcinoma (PTMC) is defined as PTC with a diameter less than 1 cm. Although the prognosis of PTC and PTMC is good, patients with advanced PTC can often be found. Skip lymph node metastasis in PTC refers to lateral cervical lymph nodes metastasis without central lymph nodes metastasis, which is a special type of lymph node metastasis (1). Skip lymph node metastasis is rare in PTC, but it affects the prognosis and recurrence of patients (2). Distant metastasis in PTC is also rare, especially the multiple distant metastasis. It occurs in about 2%–13% of patients with PTC and significantly reduces the survival rate of these patients (3). We reported a case of PTMC with skip lymph nodes metastasis and multiple distant metastasis. This case might give us a new understanding of PTMC metastasis and require us to evaluate lymph node metastasis more reasonably, especially for evaluating the skip lymph node metastasis. With the increasing incidence of thyroid cancer, the treatment of thyroid cancer should be more individualized, and elaborate preoperative investigations can help us to diagnose and treat some rare cases.

## Case presentation

**Figure 1:**
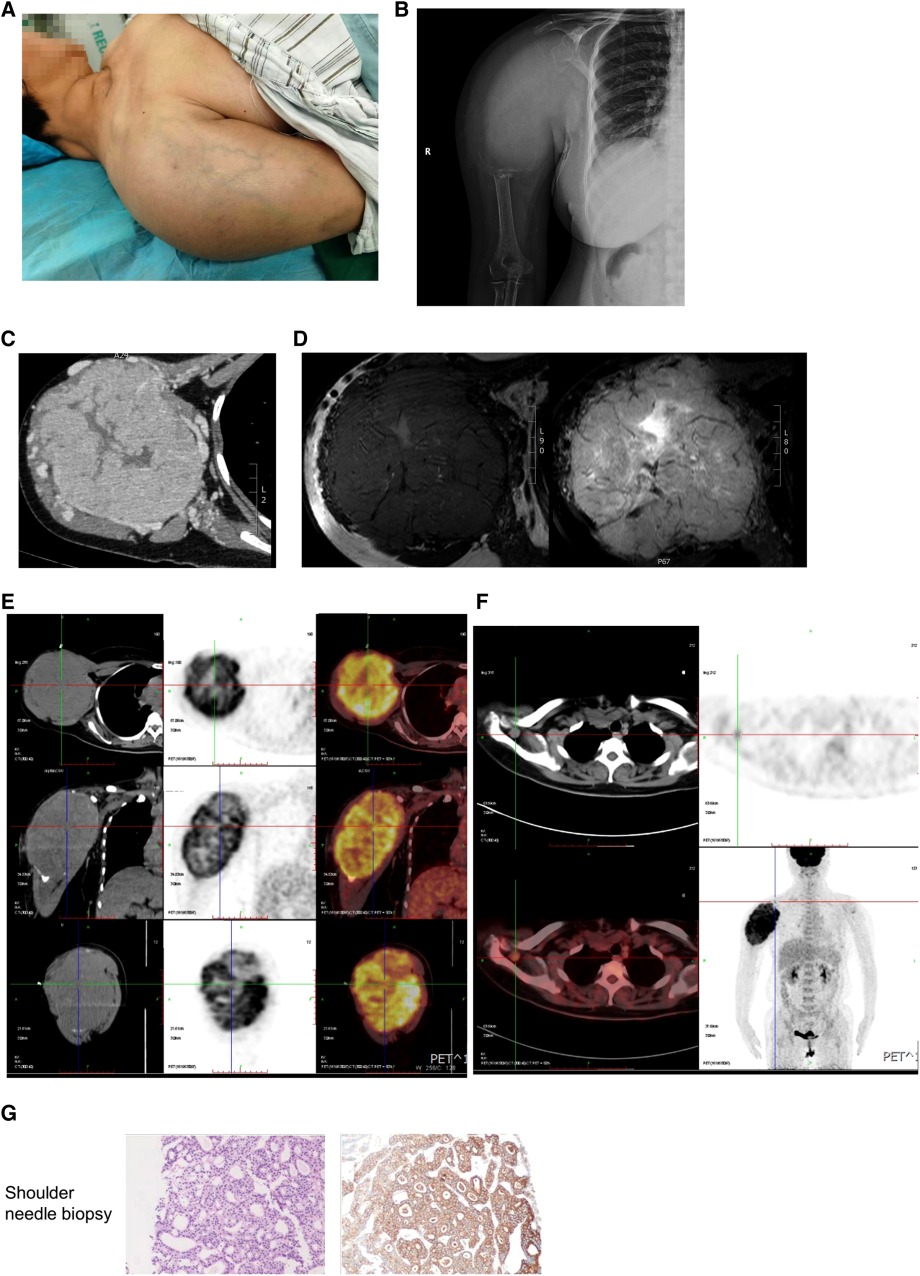
Examination and pathology of shoulder tumor. (**A**) The appearance of right shoulder. (**B**) The x-ray of the right humerus showed a vague high-density shadow with unclear boundary, and bone destruction near humerus and scapula. (**C**) Contrast-enhanced CT of the right humerus showed a soft mass replacing the normal bone structure with unclear boundary and dilated blood vessels. (**D**) No normal structure was found in the right humerus, shoulder joint and clavicle by MRI. T1 weighted image shows a mass with low signal intensity. T2 weighted image shows high signal intensity. (**E**,**F**) ^18^F-FDG PET/CT image showed that increased uptake in shoulder tumor (**E**) and the right supraclavicular fossa (**F**). (**G**) Needle biopsy of shoulder mass.

**Figure 2:**
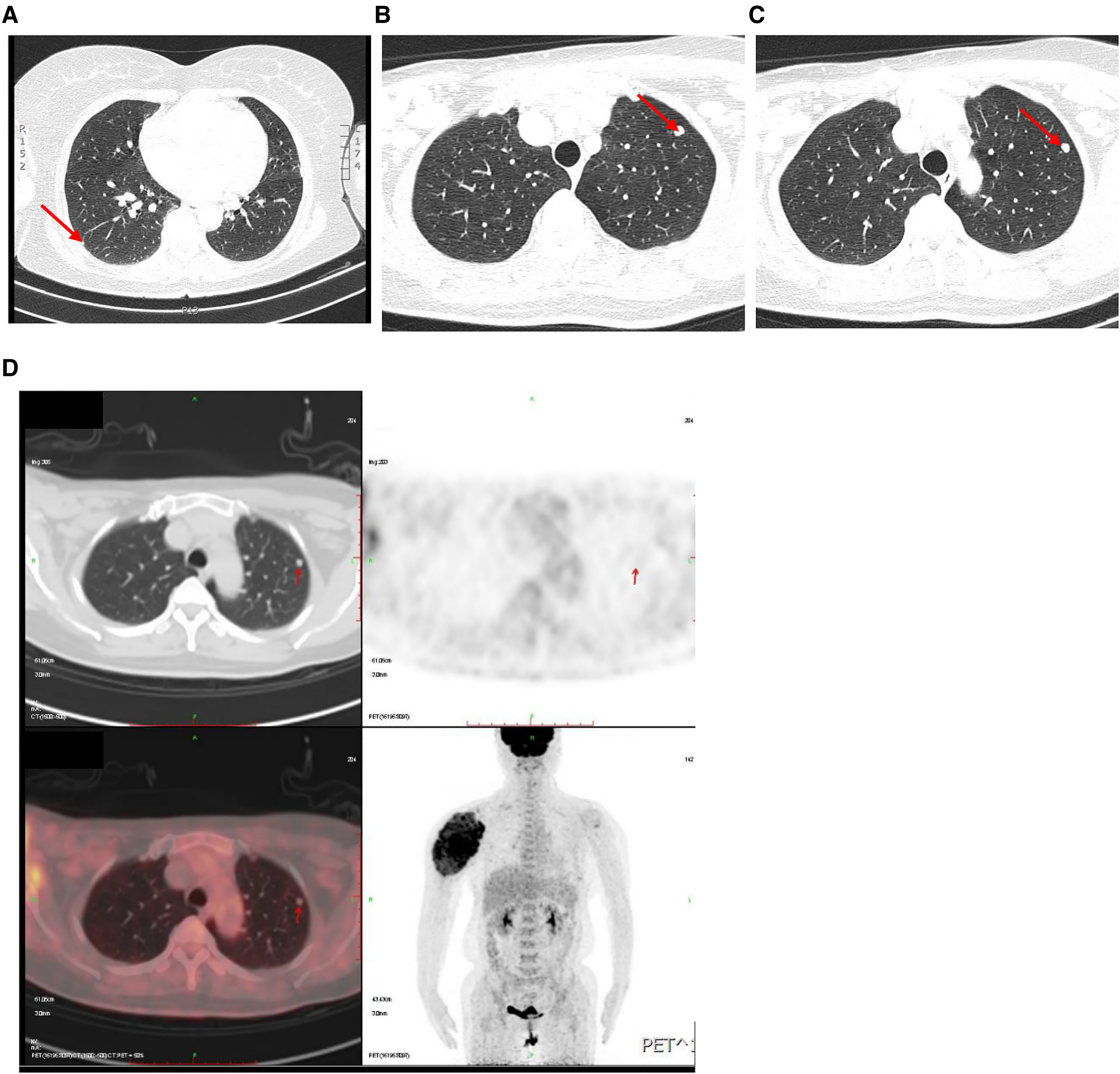
Examination of lung. (**A**–**C**) High-resolution CT of pulmonary showed multiple solidity nodules. (**D**) ^18^F-FDG PET/CT images showed increased uptake in pulmonary nodules.

A 50-year-old woman was admitted to our hospital because of pain in her right humerus for 5 years, swelling with limited movement for 4 years in February 2022. Physical Examination: a huge mass with pain could be seen in the right shoulder, which disrupted the normal shoulder. Superficial varicose veins could be seen on the surface of the mass, without swelling or ulcer. The movement of the right shoulder joint was obviously restricted (Figure 1A). Imaging examinations were conducted for the right shoulder of the patient. X-ray, CT and MRI demonstrated a huge soft tissue mass in the upper part of the right humerus, and enlarged lymph nodes in the right supraclavicular fossa, that is, the IV segment of the right lateral neck (Figures 1B–D). In addition, multiple solid nodules in both lungs were found by lung CT (Figures 2A–C). The ^18^F-FDG PET/CT image also confirmed the above results (Figures 1E,F, 2D). In order to make a definite diagnosis, the patient underwent the needle biopsy for shoulder mass. The pathological result showed that the shoulder mass was a metastatic tumor, possibly originating from thyroid *via* immunohistochemistry analysis of tissue samples with CK (+), CK7 (+), Ki-67 (10%+), TTF-1(+), TG (+), HPC (−), AFP (−), Syn (−), CgA (−), SATB2 (−), CT (−), p53 (−), NapsinA (−), ER (−), PR (−), CDX2 (−) (Figure 1G).

**Figure 3:**
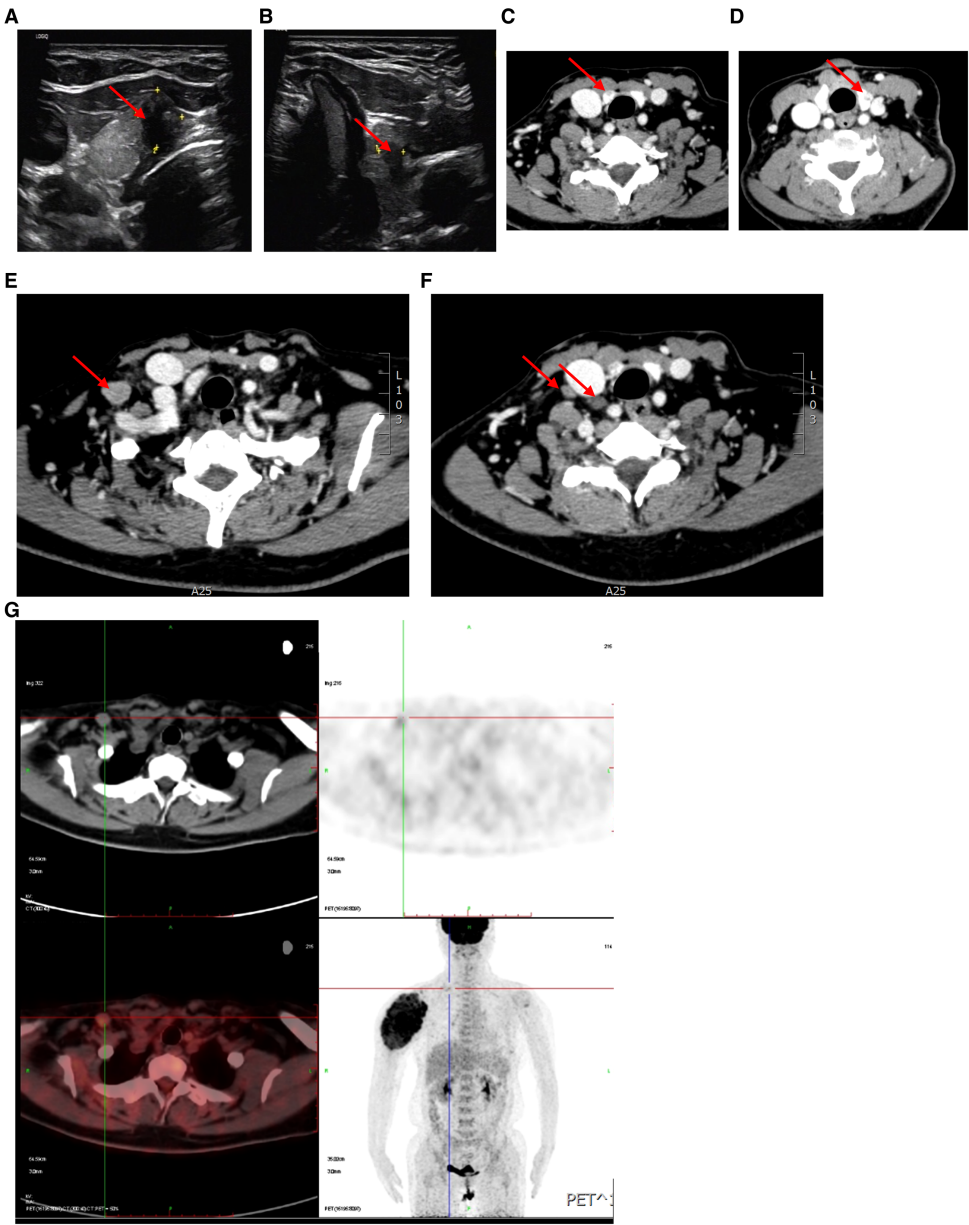
Examination of thyroid and cervical lymph nodes. (**A**,**B**) CE-US of thyroid showed within the upper portion of the left thyroid lobe was a hypoechoic, taller than wide, irregular nodule (red arrow), deeming it a TI-RADS 5. (**C**,**D**) Contrast-enhanced CT of the thyroid identified hypodense nodules within both thyroid lobes. (**E**,**F**) CT scan showed high density nodules in the right lateral cervical region. (**G**) ^18^F-FDG PET/CT images showed increased uptake in right supraclavicular fossa.

**Figure 4:**
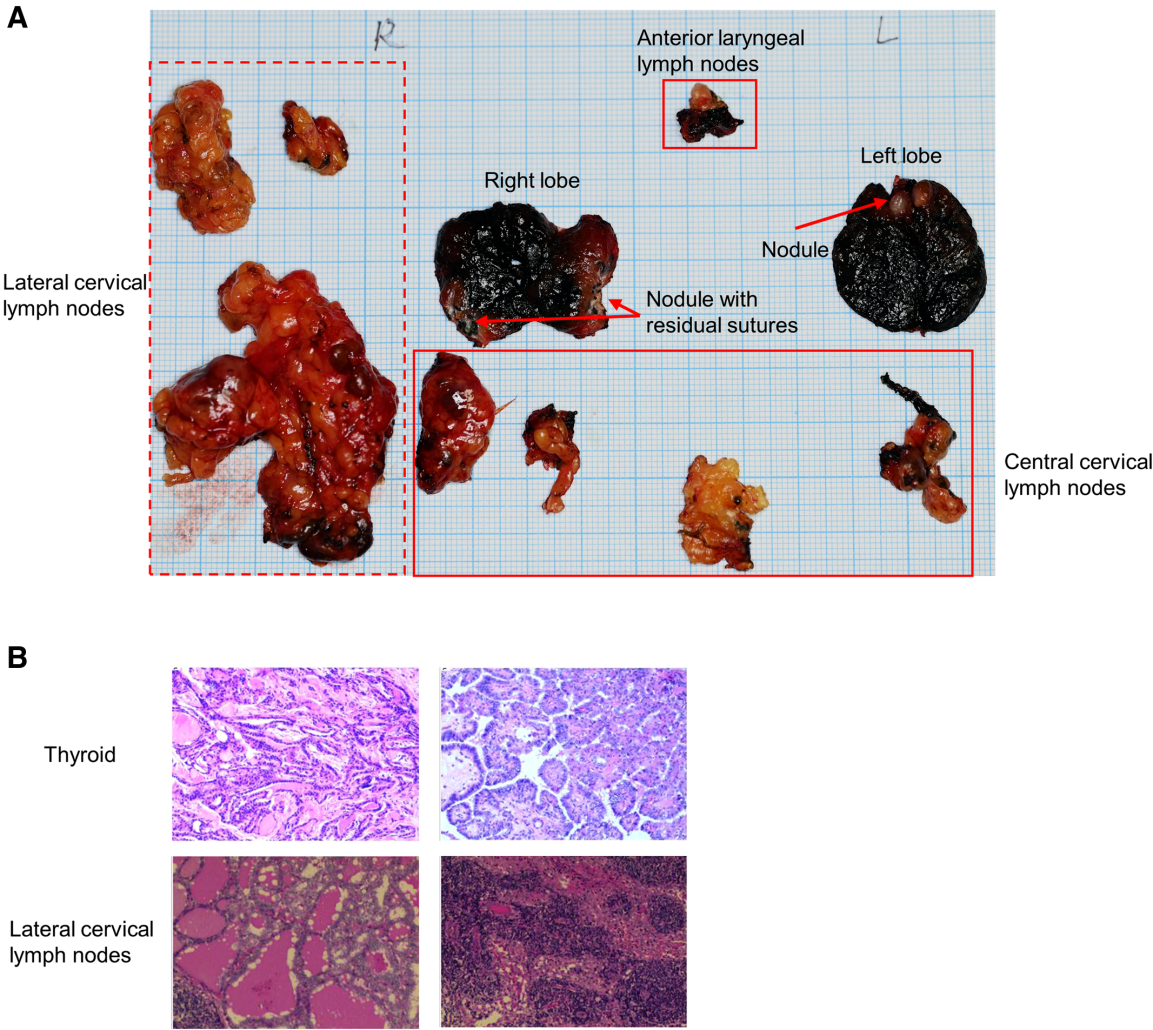
Postoperative specimen and pathology. (**A**) Thyroid and lymph nodes specimen. Dotted line box indicates lateral cervical lymph nodes, solid line box indicates central cervical lymph nodes. (**B**) Pathology of thyroid and lymph nodes.

Then, thyroid examinations were carried out. The serum free thyroxine, TSH, A-TG, A-TPO, PTH and calcitionin were all normal. It was worth noting that the serum thyroglobulin was over 10,000 ng/ml. The thyroid contrast-enhanced ultrasound (CE-US) showed 5.3 mm and 8.4 mm nodules in left and right lobes with TI-RADS 5 (Figures 3A,B). Metastasis was considered to be in the prelaryngeal lymph node (also called Delphian lymph nodes), rather than in lateral cervical lymph nodes by CE-US. The contrast-enhanced CT of thyroid also showed the nodules in thyroid (Figures 3C,D) and enlarged lymph nodes in the right supraclavicular fossa (Figures 3E,F). However, the ^18^F-FDG PET/CT scan did not find any abnormal glucose metabolism in the thyroid gland and central cervical lymph nodes, but did in the right supraclavicular fossa, which was different from CE-US (Figure 3G). Finally, total thyroidectomy, central cervical lymph node dissection and right lateral cervical lymph node dissection were performed for the patient in March 2022 (Figure 4A). Pathological results show that the nodules in both sides were classical papillary thyroid microcarcinoma (Figure 4B). The BRAF-V600E of the patient was wild-type. Additionally, sutures were found in the cancerous nodule in the right lobe, which came from a partial thyroidectomy for a thyroid benign nodule in 2012 (Figure 4A). Interestingly, the lymph nodes suspected of metastasis by CE-US did not show metastasis, while the lateral lymph nodes showed metastasis.

## Discussion

The incidence of skip lymph node metastasis and distant metastasis in PTC is very low. It is significantly related to the prognosis and recurrence of the patients. Thus, the mentioned patients should be carefully screened, and personalized and collaborative multi-disciplinary care should be given. In this case, the patient who had PTMC with skip lymph node metastasis, huge shoulder metastasis and lung metastasis is very rare. By screening the diagnosis and treatment process, we can have new views on this kind of patients. We will discuss some new insights from the following three angles, including skip lymph node metastasis with inconsistent preoperative results, rare humeral metastasis, and malignant change around residual sutures from previous surgery.

### Skip lymph nodes metastasis

Skip lymph nodes metastasis is a special type of lymph node metastasis, which usually refers to lateral cervical lymph node metastasis without central lymph node metastasis (1). The incidence of skip metastases is about 3%–19.7% (2). Generally, the non-invasive examinations used to evaluate lymph node metastasis are imaging examinations, such as ultrasound and CT.

Besides, molecular markers can also be used to predict the lymph node metastasis. TERT promoter mutations and RET/PTC rearrangements were associated with distant metastasis (4–6). Interestingly, BRAF mutations were found to be not related with distant metastasis in PTC (5). Another study found that 25-genes could distinguish N0 and N1 in PTC (7). Additionally, plasma N-glycomics, microRNA-222 and ANGPTL1 were all proved to be biomarkers for predicting lymph node metastasis of PTC (8–10).

In our case, the enlarged prelaryngeal lymph nodes were suspected of metastasis by the thyroid CE-US. ^18^F-FDG PET/CT image did not show any abnormal glucose metabolism in the thyroid and cervical lymph nodes, but found in the right supraclavicular fossa. The pathological results of the patient demonstrated that there was skip lymph node metastasis in right lateral cervical region IV, which was inconsistent with the thyroid CE-US. Therefore, we need a more effective evaluation method for skip lymph node metastasis. Lee proposed that the location of lymphatic metastasis was related to the location of nodules in the thyroid gland (11). Another study on skip metastasis in PTC demonstrated that thyroid capsular invasion, multifocality, tumor in the upper portion, and maximum tumor diameter ≥ 1 cm were independent risk factors (12). For suspected metastatic lymph nodes, fine needle aspiration cytology (FANC) combined with the thyroglobulin might be useful (1). For the primary patients with high-risk factors, the lateral cervical lymph nodes should be carefully evaluated to reduce the postoperative recurrence. The research of Hao Fu demonstrated the value of PET/CT in evaluation of lymph nodes and distant metastasis in PTC patients. And the diagnosis performance of gallium 68-labeled fibroblast activation protein inhibitor PET/CT was superior to fluorine 18 ﬂuorodeoxyglucose PET/CT, which we could consider to utilize in advanced PTC patients in further clinical practice (13).

### Distant metastasis

The incidence of distant metastasis of PTC is less than 5%. The main sites of distant metastases are the pulmonary and bone, which leads to a significant decline in survival rates (14).

Pulmonary metastasis in PTC is rare. Previous studies found that male, old age, large tumor, extrathyroidal extension and lymph node metastasis were related to pulmonary metastasis (15, 16). Additionally, bilateral lateral lymph node metastasis was also an important risk factor (15). In our case, this patient with the right lateral cervical lymph node metastasis developed pulmonary metastasis. Therefore, lung CT should be included in the preoperative examination, when lateral lymph node metastasis is suspected.

Bone is the second most common metastatic site in PTC, and the mechanism of bone metastasis may be different in different patients. The incidence of bone metastasis is about 2%–13% (3). Bone metastasis mainly occurs in the spine (34.6%), followed by the pelvis (25.5%) (17). Bone metastasis is very insidious. Pain and fracture are the most common clinical manifestations. The most convenient method to evaluate bone metastasis is imaging examinations, including x-ray, CT and MRI (18).

In our case, the patient showed obvious pain and swelling in her right shoulder. First of all, we considered that it was a bone tumor. However, the pathological results showed that it originated from thyroid carcinoma. Although this patient suffered with bone metastasis in the upper region of the right humeral, the site was different from the traditional site where bone metastasis occurred. This might be related to risk factors of the patient, especially the skip lymph node metastasis in the lateral cervical region. Recent studies showed that larger tumor size (>4 cm), extrathyroidal extension and lymph node metastasis were independent prognostic factors for bone recurrence. Moreover, large lymph node metastasis was significantly related to bone metastasis. Ito et al. found that lymph node metastases larger than 3 cm was a significant predictor of bone recurrence and also predicted a poor prognosis (19).

In conclusion, early detection for distant metastasis is closely related to the patients’ prognosis and survival. Besides, lateral lymph node metastasis plays an important role in distant metastasis.

### Malignant changes at residual sutures

The patient reported in this case underwent surgery for a benign thyroid nodule in the right lobe of the thyroid 10 years ago, and residual sutures were seen in the right nodule during this surgery. Unfortunately, pathological results showed that the small lesion around the sutures was PTMC. The malignant nodule was suspected to be related to a long-term inflammatory response caused by residual sutures. There are many reports revealing the relationship between inflammation and postoperative tumor recurrence (20). A study demonstrated that the prognostic score of inflammation was related to distal extrahepatic bile duct cancer after pancreaticoduodenectomy. Further studies have shown that the prognostic score of inflammation was an independent risk factor for recurrence of distal extrahepatic bile duct cancer (21).Thus, inflammation caused by residual sutures is also an influential factor which should not be ignored. In thyroid surgery, we can use the ultrasonic scalpel to hemostasis, and try to avoid using non-absorbable sutures to reduce inflammation caused by suture residues.

### Limitations

In the study, we reported a rare case of PTMC with skip lymph node metastasis and multiple distant metastasis. There are some limitations in the case. First, we didn’t perform iodine scan and radioactive iodine ablation for this patient. Although MDT was performed on the patient, the patient was unwilling to undergo amputation, which might significantly affect the curative effect. Moreover, we developed the treatment plan through MDT, but the patient did not return after discharge due to her own problems, and result in lacking of follow-up. Second, the lung nodules were not certainly diagnosed as PTC metastasis, but they were highly suspicious. In addition, only BRAF was detected for the patient, and TERT was not detected. Therefore, a more complete examination and follow-up may be more helpful for our understanding of this patient.

Thyroid cancer is an inert tumor with good prognosis, and conventional surgery is suitable for most patients. However, a small proportion of patients may be suffered from skip lymph node metastasis and distant metastasis. For this kind of patients, interdisciplinary consultation for diagnosis and treatment are needed to reduce the risk of recurrence, improve survival rate and improve the quality of life.

## Data Availability

The original contributions presented in the study are included in the article/Supplementary Material, further inquiries can be directed to the corresponding author/s.
